# Implementation of Revised Simplified Geneva Score in Triage Nurse Evaluation for Patients With Suspected Pulmonary Embolism: A Retrospective Chart Review

**DOI:** 10.1155/emmi/2807776

**Published:** 2025-05-16

**Authors:** Nicola Osti, Alberto Maino, Giulia Moreschini, Cristina Marinconz, Nicola Susca, Cristina Contu, Vito Racanelli, Anna Brugnolli

**Affiliations:** ^1^Department of Medicine, Azienda Provinciale per i Servizi Sanitari (APSS), Trento, Italy; ^2^Center for Medical Sciences (CISMed), University of Trento, Trento, Italy

## Abstract

**Background:** Pulmonary embolism (PE) is a clinical condition frequently encountered in the emergency department (ED), with a high and early mortality rate. ED triage determines the priority of further evaluation of care at the time of patient arrival. Very little is known about the specific role of ED triage in PE. We aimed to evaluate (1) whether the current five-level triage (5LT) system can identify patients with PE and differently prioritize them for medical evaluation and (2) the discriminatory capacity of simplified revised Geneva score (SRGS) toward PE diagnosis when calculated by triage nurses.

**Methods:** A retrospective chart review on ED patients who underwent computed tomography pulmonary angiography (CTPA) in 2023. Based on the CTPA report, patients were categorized into two subgroups: CTPA PE-negative and CTPA PE-positive. We then searched for correlations between PE diagnosis and triage priority level, time from triage to medical evaluation, SRGS, and National Early Warning Score 2 (NEWS2).

**Results:** Of the 196 patients included in the analysis (age 71.1 ± 16.9), 45 (23.0%) were CTPA PE-positive (26 proximal PE and 19 distal PE). There was no correlation between the assigned triage color code and the CTPA results. Although we found a statistically significant difference in the prevalence of CTPA-confirmed PE according to the results of the SRGS (*p* = 0.014), the SRGS calculated at the time of triage showed a poor prediction accuracy for subsequent PE diagnosis (area under curve [AUC] 0.608). NEWS2 was significantly associated with the triage-assigned priority level (*p* < 0.001).

**Conclusions:** The current 5LT was unable to differently prioritize patients with or without PE, and it seems unlikely that implementation of SRGS in the triage nurse evaluation will significantly improve the prioritization of patients with suspected PE for medical evaluation. Nonetheless, application of SRGS in triage evaluation may improve the appropriateness of the subsequent clinical pathway for PE diagnosis and risk stratification.

## 1. Introduction

Venous thromboembolism, behind myocardial infarction and stroke, currently represents the third cause of cardiovascular morbidity and mortality [1].

A recent Canadian observational study estimated for pulmonary embolism (PE) an incidence rate of 0.35 per 1000 persons/year (95% CI 0.19–0.50), with female sex and advanced age representing well-known risk factors [2]. In a recent report by Hsu et al. based on the National Hospital Ambulatory Medical Care Survey, the proportion of ED visits for PE increased from 0.1% in the 2010–2012 period to 0.2% in the 2017–2018 period [3]. Mortality ranges from less than 1% in lower risk categories to about 20%–25% in higher risk categories [4].

According to 2019 European Society of Cardiology (ESC) guidelines for the diagnosis and management of acute PE, PE diagnosis should be based on a Bayesian approach that starts with the assessment of the clinical probability of PE [5].

Clinical probability can be assessed by clinical judgment or by the implementation of clinical prediction rules (CPRs), such as the revised Geneva score (RGS) or Wells score [5]. The performance of the different CPRs proposed in the current medical literature is comparable [6]. Pretest probability guides the testing strategy and interpretation of test results, reduces patients' exposure to radiological examinations, and avoids PE overdiagnosis and the associated costs [7, 8].

ED triage is the process of quickly sorting patients to determine the priority of further evaluation of care at the time of patient arrival in order to assign the right resources to the right patient in the right place at the right time [9]. According to the Manchester Triage System (MTS), the assessment is performed immediately after the arrival of the patient and leads to the assignment of a color code that corresponds to a time window within which a first physician contact should occur. Very little is known about the capability of the MST to correctly stratify patients with suspected PE at the moment of their arrival at ED, and the employment of a CPR specific for PE is currently not advised.

We conducted a pilot analysis on patients who underwent a computed tomographic pulmonary angiography (CTPA) in the ED in order to evaluate whether the clinical probability and severity of PE could be predicted at triage, with the implementation of simplified revised Geneva score (SRGS) and the National Early Warning Score 2 (NEWS2), respectively.

## 2. Methods

### 2.1. Study Design

We performed a retrospective chart review on patients who underwent CTPA in the ED of a regional Hospital in Northern Italy from 1st January 2023 to 31st December 2023.

### 2.2. Study Population

Data were collected from the electronic health records of outpatients older than 18 years admitted to the Emergency Department from 1st January 2023 to 31st December 2023. Patients were identified on the basis of the CTPA report as CTPA-positive or CTPA-negative for PE. For subgroup analyses, we also distinguished between patients with distal PE (segmental or subsegmental) and proximal PE (lobar or main pulmonary arteries).

### 2.3. Setting

Triage was performed by a trained nurse following the local triage protocol derived from the 2019 Italian Ministerial Guidelines on ED Triage [10], which provides for adult patients 27 different flowcharts according to the main clinical problem at presentation. Five priority code levels (5LT) were provided for each main clinical problem: red for emergency, orange for undeferrable urgency, blue for deferrable urgency, green for minor urgencies, and white for nonurgent situations. As an example, Table 1 reports the flowcharts used for dyspnea and nontraumatic chest pain, representing the two main clinical presentation of PE. The use of PE-specific risk rating scales was not provided.

D-dimer was requested by the triage nurse or by the physician based on personal clinical judgment, and CTPA was generally performed in the presence of a positive result (D-dimer > 500 mcg/dL). In the presence of a high clinical suspicion, CTPA was directly requested by the physician without prior D-dimer testing.

### 2.4. Data Collection and Instrument

The following demographic and clinical data were collected from the electronic medical records: age, sex, heart rate, blood pressure, and peripheral oxygen saturation at time of triage evaluation; personal anamnestic data (active cancer, recent surgery of fracture [< 30 days], presence of chronic cardio-pulmonary diseases, and hemoptysis at time of presentation); presence of clinical signs and symptoms of lower limbs DVT; D-dimer levels (when performed).

Color code assigned by the triage nurse was also collected, along with the time needed for medical evaluation, total time spent in the emergency room, and the 30-day mortality.

Data were used for descriptive analyses and to retrospectively calculate the SRGS and the NEWS2. NEWS2 is a validated track and trigger system to assess illness severity and risk of deterioration for patients in acute care settings.

The SRGS was chosen instead of the Wells score because it is more standardized and less dependent on personal clinical judgment; it is also more suitable for an Italian context since it is validated on a large European population.

SRGS and NEWS2 are summarized in Tables 2 and 3.

### 2.5. Statistical Analysis

Distributions of continuous variables were expressed as mean and standard deviation.

Skewed continuous variables, including D-dimer levels, time needed for medical evaluation, and total time spent in the ED, were logarithmically transformed, and then geometric means with 95% confidence intervals (CIs) were reported.

Categorical variables were expressed as proportions.

Student's *t*-test was used to compare the means between two groups, whereas ANOVA was used to compare the means among three groups. Qualitative data were analyzed by *χ*^2^ test or *χ*^2^ test for linear trend analysis when indicated.

The evaluation of the prediction accuracy was obtained by calculating the sensitivity (SN), specificity (SP), positive predictive value (PPV), and negative predictive value (NPV), with representation of the relative area under the curve (AUC) in a receiver operating characteristic (ROC) analysis.

Statistical analysis was performed using the IBM SPSS 23.0 statistical package (IBM Inc., Armonk, USA).

### 2.6. Ethics Approval of the Study

The study design did not reveal any ethical problems. Processing of sensitive data was performed only through limited access by the researcher. Since data were evaluated retrospectively and solely for epidemiological purposes, written informed consent was not deemed necessary. We obtained permission from the Hospital Medical Direction for data collection and analysis.

## 3. Results

Approximately 25,000 patients visited the named ED in 2023. Of those, CTPA was requested in 198 cases. Two patients were excluded because CTPA provided inconclusive results due to the lack of iodinated contrast medium. Therefore, a total of 196 patients were included in the analysis.

Table 4 summarizes the main demographic and clinical characteristics of the patients included. Mean age was 71.1 ± 16.9 years; male and female sexes were equally distributed (respectively, 50.5% and 49.5%).

The three main symptoms at the presentation that prompted the CTPA request were dyspnea, chest pain, and syncope. For 167 patients (85.2%), a D-dimer test was requested before CTPA. CTPA yielded a positive result in 45 out of 196 patients (23.0%), of which 19 were distal PE and 26 were proximal PE.

Table 5 summarizes age distribution, sex, time to medical evaluation, time spent in the ED, CTPA report, SRGS class of risk, NEWS2 class of risk, and D-dimer levels according to the assigned triage color code. Patients with the highest priority levels had shorter times to medical evaluation (mean 13 [95% CI 9–19] minutes for red, 22 [95% CI 20–25] minutes for orange, 62 [95% CI 51–75] minutes for blue, 66 [95% CI 42–105] minutes for green, *p* < 0.001) and also shorter time spent in the ED (mean 271 [95% CI 220–333] minutes for red, 326 [95% CI 303–350] minutes for orange, 354 [95% CI 318–395] minutes for blue, 401 [95% CI 349–461] minutes for green, *p* = 0.006). We were unable to find an association between the assigned triage color code and the PE diagnosis made by CTPA, either when distal PE was considered separately from proximal PE (*p* = 0.417) or when it was considered together (*p* = 0.954).

NEWS2 showed a strong association with the triage color code (*p* < 0.001); 8.8% of the patients assigned the highest priority level could be defined as “high risk” of clinical deterioration and mortality according to NEWS2 results. Fifteen out of 196 patients (7.7%) died within one month from ED admission, and NEWS2 showed a strong association with 30-day mortality (*p* < 0.001).

Patients assigned to the highest triage priority levels were also older and had higher D-dimer levels than patients assigned to the lowest priority levels.

Results of the distribution of age, sex, time to medical evaluation, time spent in the ED, SRGS, SRGS class of risk, NEWS2 and D-dimer levels according to the CTPA report are shown in Table 6.

There were no differences in the time needed for medical evaluation according to the presence or absence of PE (mean 33 (95% CI 29–38) minutes for CTPA PE-negative patients, 28 (95% CI 31–37) minutes for CTPA PE-positive patients, *p* = 0.403) nor in the total time spent in the ED (mean 338 (95% CI 317–360) minutes for CTPA PE-negative patients, 324 (95% CI 285–367) minutes for CTPA PE-positive patients, *p* = 0.577).

A statistically significant difference emerged for the prevalence of CTPA-confirmed PE according to the results of the SRGS, both when patients with subsegmental PE were included in the analysis (median 2 (1–3) for CTPA PE-negative and 2 (2–3.5) for CTPA PE-positive, *p* = 0.011), excluded (*p* = 0.007) or considered together with patients with proximal PE (*p* = 0.014).

Statistical significance was lost (*p* = 0.152) when patients (with or without PE) were stratified into a three-level probability model according to the results of the SRGS (low probability 0–1; intermediate probability 2–4; high probability ≥ 5). According to this model, for patients identified as low probability (SRGS 0–1), a negative CTPA could exclude PE at triage with a NPV of 86.2%.

**Figure 1:**
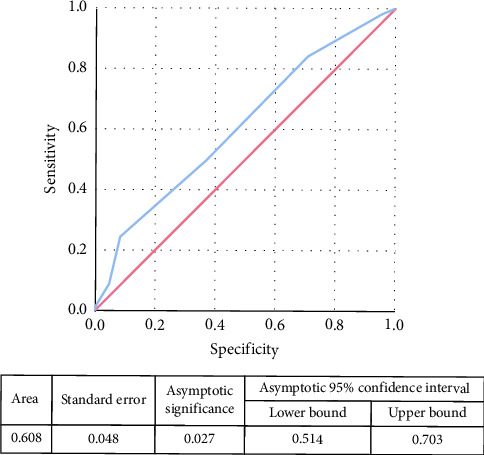
Receiver operating characteristic (ROC) curve for the association between simplified revised Geneva score (SRGS) and pulmonary embolism (PE) diagnosis made by computed tomography pulmonary angiography (CTPA).

Figure 1 represents the ROC curve for the association between SRGS calculated at triage and subsequent PE diagnosis made by CTPA.

## 4. Discussion

PE is a clinical condition characterized by early mortality, and therefore, prompt suspicion and recognition are of paramount importance in the ED. To the best of our knowledge, no PE-specific triage algorithm is described in the current medical literature.

A previous study demonstrated the capability of the MTS to prioritize patients with PE, but the study was focused on high-risk patients and did not include a control group when analyzing times for medical evaluation [11].

In our study, the current 5LT prioritization system alone was not able to prioritize patients with or without PE differently, nor did patients with confirmed PE have shorter times for medical evaluation, both when patients with subsegmental PE were included or excluded in the analysis.

Regarding the severity of PE at presentation, the vast majority of patients with a high risk of clinical deterioration, according to the NEWS2, independently from PE diagnosis, were correctly recognized as a high priority; a systematic calculation of NEWS2 at triage will be therefore time-consuming without a clinical benefit.

The relatively small number of patients with confirmed PE did not allow us to investigate a linear association between triage color code and Pulmonary Embolism Stratification Index (PESI) [12] which is currently recommended by the ESC guidelines to stratify the risk of early clinical deterioration and mortality of patients with confirmed PE. Besides, PESI score has been studied and validated for patients with a confirmed diagnosis of PE, which is not the case of ED triage where diagnosis is only presumptive.

Triage function is of paramount importance for both patient safety and health system functioning, with both over- and undertriage implying risks when systems are overstretched due to excessive patients' affluence. For example, patients assigned with minor urgency codes, but having instead a potentially life-threating condition that has been misrecognized at triage evaluation, could spontaneously leave the ED due to long waiting times without being seen [13].

Very often triage nurses rely on their personal gestalt to make clinical hypotheses that are clearly necessary to give the appropriate priority code for medical evaluation; in this sense, having a rapid and reproducible tool with good discriminatory capacity could result in a net clinical benefit.

For example, Zaboli et al. demonstrated better accuracy of the Emergency Department Assessment of Chest Pain Score (EDACS) compared to personal clinical judgment when applied by triage nurses to assess the priority level of patients presenting with acute chest pain [14].

With regard to PE, the application of the RGS in the ED in patients presenting with chest symptoms has been proposed by Le Gal and Bounameaux [15], who found PE diagnosis for a NPV of 0.993 in case of low clinical probability according with RGS, but the number of PE included in the study was very small (11 PE out of 1002 total patients).

From our analysis emerged also for the SRGS a good NPV in patients with low clinical probability for PE (86.2%), but the prediction accuracy of the SRGS calculated at triage for subsequent PE diagnosis was poor (AUC 0.608).

It seems therefore improbable, in agreement with our results, that implementation of the SRGS in the triage evaluation could significantly ameliorate the prioritization of patients with suspected PE for medical evaluation since, in our study, none of the patients with a high SRGS (defined as ≥ 5 according to a three-level pretest probability model [5]), and none of the patients with proximal PE were identified with a low priority color code. Indeed, the four patients who were given a low priority code (green) by triage nurse had distal PE.

On the other hand, in our experience, implementation of the SRGS at triage can ameliorate the appropriateness of PE diagnosis. In our ED, as well as in many other working situations around the world [7, 8], the increasing availability of CTPA has led to a tendency to suspect PE much more frequently than before, and to incorrectly request D-dimer in an extremely high proportion of patients presenting with chest symptoms or syncope.

This overall tendency is well illustrated by the decreasing percentage of PE confirmation after its clinical suspicion in the last decades; incidences as low as 5% have been reported in some studies, in strong contrast with nearly 50% incidence reported in the early 1980s [16].

### 4.1. Limitations

The major bias of our study is the asymmetry between the two main study groups (CTPA positive and CTPA negative) since data were collected from a period in which adherence to the ESC 2019 diagnostic algorithm was poor.

In second place, the number of patients included is still pretty low, and some lacks of statistical significance are due to the small numbers of the subgroups.

We also performed subgroup analysis for patients with proximal and distal PE since very little is known about the clinical outcomes of patients presenting distal isolated emboli at CTPA [16], but the sample size was too small to obtain consistent data.

Another important limitation of our study is that clinical scores have been retrospectively reconstructed and could, therefore, slightly differ from the same score when calculated in real time; in particular, the retrospective evaluation of signs and symptoms of DVT might have introduced a misclassification bias in the SRGS calculation.

Finally, data collection was conducted in proximity to the COVID-19 pandemic. Nevertheless, its influence on the results is unlikely, given the fact that in the 40 patients in which a nasopharyngeal swab for SARS-CoV-2 infection was requested, only six yielded positive results.

## 5. Conclusions

The current 5LT was unable to differently prioritize patients with or without PE, and it seems unlikely that implementation of SRGS carried out by the triage nurse will significantly improve the prioritization of patients with suspected PE for medical evaluation. Nonetheless, application of SRGS in triage evaluation may improve the appropriateness of the subsequent clinical pathway for PE diagnosis and risk stratification, since highlighting at triage evaluation the clinical (pretest) probability for PE could lead to a more appropriate D-dimer request and therefore a more appropriate CTPA request. We therefore believe that the introduction of disease-specific CPRs and other tools for patients' risk stratification in the triage evaluation is an issue of paramount importance, and studies with large sample sizes and high methodological quality are needed to better define the benefits of their application in daily clinical practice.

## Figures and Tables

**Table 1:** Triage flowchart for dyspnea and nontraumatic chest pain according to the current local protocol.

Dyspnea	Nontraumatic chest pain
**Red: emergency**	

SpO < 90% in patients without chronic pulmonary diseases	SpO < 90% in patients without chronic pulmonary diseases
SBP < 90 mmHg	SBP < 90 mmHg
HR < 40 bpm or > 200 bpm	HR < 40 bpm or > 200 bpm
RR < 7 per minute or > 30 per minute	RR < 7 per minute or > 30 per minute
P or U on AVPU scale	P or U on AVPU scale
Temperature < 34°C or > 41°C	Temperature < 34°C or > 41°C
Airways obstruction or gasping	STEMI
Severe dyspnea with chest pain	
Severe dyspnea with allergic reaction	

**Orange: undeferrable urgency**	

SpO 90%–94% in patients without chronic pulmonary diseases	Ongoing acute chest pain (cardiac or cardiac) within 6 h from onset
RR 25–30 bpm	Chest associated with syncope
V on AVPU scale	Chest pain associated with palpitations
Cyanosis, tirage, or orthopnea	Chest pain associated with dyspnea
Signs or clinical suspicion of DVT	Recent cocaine use
Associated abdominal pain or diaphoresis	

**Blue: deferrable urgency**	

Temperature > 38°C	Acute chest pain (cardiac or cardiac) regressed from more than 6 h, with normal vital parameters and with positive risk factor for ACS
Asthma without amelioration after self-administered therapy	Acute chest pain (cardiac or cardiac) regressed from more than 6 h, with normal vital parameters and with positive risk factor for PE

**Green: minor urgency**	

Reported dyspnea in patients with normal vital parameters and no chronic cardiopulmonary diseases	

**White: nonurgent**	

Nonurgent classification discouraged	Nonurgent classification discouraged

*Note:* AVPU scale: alert: the patient is awake and responsive; verbal: the patient responds to verbal commands; pain: the patient responds to painful stimuli; unresponsive: the patient does not respond to verbal or painful stimuli.

Abbreviations: ACS, acute coronary syndrome; DVT, deep vein thrombosis; HR, heart rate; PE, pulmonary embolism; RR, respiratory rate; SBP, systolic blood pressure; SpO2, peripheral oxygen saturation STEMI, ST elevation myocardial infarction.

**Table 2:** Simplified revised Geneva score (SRGS).

Items	Points
Previous PE or DVT	1
Heart rate	
- 75–94 b.p.m.	1
- ≥ 95 b.p.m.	2
Surgery or fracture within the past month	1
Hemoptysis	1
Active cancer	1
Unilateral lower-limb pain	1
Pain on lower-limb deep venous palpation and unilateral edema	1
Age > 65 years	1
The score obtained relates to probability of PE:	
0–3 points low probability (8%)	
4–10 points intermediate probability (29%)	
≥ 11 points high probability (74%)	

Abbreviations: b.p.m., beats per minute; DVT, deep vein thrombosis; PE, pulmonary embolism.

**Table 3:** National early warning Score 2 (NEWS2).

Physiological parameter	Score						
	3	2	1	0	1	2	3
Respiratory rate (per minute)	≤ 8		9–11	12–20		21–24	≥ 25
SpO2 Scale 1 (%)	≤ 91	92–93	94–95	≥ 96			
SpO2 Scale 2 (%)	≤ 83	84–85	86–87	88–92 ≥ 93 on air	93–94 on oxygen	95–96 on oxygen	≥ 97 on oxygen
Air or oxygen?		Oxygen		Air			
Systolic blood pressure	≤ 90	91–100	101–110	111–219			≥ 220
Pulse (per minute)	≤ 40		41–50	51–90	91–110	111–130	≥ 131
Consciousness				Alert			CVPU
Temperature (°C)	≤ 35.0		35.1–36.0	36.1–38.0	38.1–39.0	≥ 39.1	
	0–4 low risk						
	5-6 medium risk						
	≥ 7 high risk						

*Note:* SpO2 peripheral saturation of oxygen. CVPU, confusion, response to verbal stimuli, response to painful stimuli, and unresponsive.

**Table 4:** Main characteristics of the sample included in the analysis (N 196).

Demographics charateristics	
Age (years) ± SD	71.1 ± 16.9
Male sex	99 (50.5%)
Reason for ED access	*N* (%)
Dyspnea	86 (43.9)
Chest pain	54 (27.6)
Syncope	18 (9.2)
Suspected DVT	8 (4.1)
Palpitations	6 (3.1)
Other	24 (12.2)
Clinical disease	
Cancer	37 (18.9)
Chronic cardiopulmonary disease	66 (33.7)
Distribution of assigned triage priority levels	
Red	22 (11.2)
Orange	94 (48.0)
Blue	59 (30.1)
Green	21 (10.7)
CTPA report	
Negative	151 (77.0)
Positive	45 (23.0)
Confirmed DVT at duplex ultrasound	11 (5.6)
Vital signs	
Heart rate ≥ 110 b.p.m.	34 (17.3)
Systolic BP < 100 mmHg	12 (6.1)
Arterial oxyheamoglobin saturation < 90%	25 (12.8)
National Early Warning Score (NEWS2) score	
0–4 low risk	142 (72.4)
5-6 medium risk	22 (11.2)
≥ 7 high risk	32 (16.3)

Abbreviations: b.p.m., beats per minute; CTPA, computed tomographic pulmonary angiography; DVT, deep vein thrombosis; ED, emergency department; NEWS2, National Early Warning Score 2; SD, standard deviation.

**Table 5:** Distribution of age, sex, time to medical evaluation, time spent in the ED, CTPA report, SRGS, NEWS2, and D-dimer levels according to the triage color code.

	Total (*N* = 196)	Green (*N* = 21)	Blue (*N* = 59)	Orange (*N* = 94)	Red (*N* = 22)	*p* ^∗^
Age (years) ± SD	71.1 ± 16.9	59.7 ± 20.2	69.3 ± 16.6	72.5 ± 16.4	80.8 ± 8.4	< 0.001
Male sex (%)	50.5	47.6	45.8	53.2	54.5	0.397
Time to medical evaluation (minutes; 95% CI)	32 (28–36)	66 (42–105)	62 (51–75)	22 (20–25)	13 (9–19)	< 0.001
Time spent in ED (minutes; 95% CI)	335 (301–354)	401 (349–461)	354 (318–395)	326 (303–350)	271 (220–333)	0.006
CTPA report (*n*, %)						0.954
- Negative	151 (77.0)	17 (80.9)	46 (78.0)	71 (75.5)	17 (77.3)	
- Positive	45 (23.0)	4 (19.1)	13 (22.0)	23 (24.5)	5 (22.7)	
SRGS (*n*, %)						0.298
- Low risk (0–1)	50 (25.5)	5 (23.8)	19 (32.2)	23 (24.5)	3 (13.6)	
- Intermediate risk (2–4)	135 (68.9)	16 (76.2)	34 (57.6)	68 (72.3)	17 (77.3)	
- High risk (≥ 5)	11 (5.6)	0 (0)	6 (10.2)	3 (3.2)	2 (9.1)	
NEWS2 (*n*, %)						< 0.001
- Low risk (0–4)	142 (72.4)	21 (100)	55 (93.2)	65 (69.1)	1 (4.5)	
- Medium risk (5–6)	22 (11.2)	0 (0)	4 (6.8)	15 (16.0)	3 (13.6)	
- High risk (≥ 7)	32 (16.3)	0 (0)	0 (0)	14 (14.9)	18 (81.8)	
D-dimer (mcg/dL)	1972 (1679–2315)	1356 (997–1882)	1641 (1278–2108)	2003 (1609–2494)	4405 (1843–10527)	0.003

Abbreviations: CI, confidence interval; CTPA, computed tomography pulmonary angiography; ED, emergency department; NEWS2, National Early Warning Score 2; PE, pulmonary embolism; SRGS, simplified revised Geneva score.

^∗^ *p* value by ANOVA or *χ*^2^ for linear trends when indicated.

**Table 6:** Distribution of age, time to medical evaluation, time spent in the ED, SRGS, NEWS2, and D-dimer levels according to CTPA report.

	Total (*N* = 196)	CTPA PE-negative (*N* = 151)	CTPA PE-positive (*N* = 45)	*p* ^∗^
Age (years ± SD)	71.1 ± 16.9	70.3 ± 16.8	73.7 ± 17.1	0.240
Male sex (%)	50.5	49.7	53.3	0.186
Time to medical evaluation (minutes; 95% CI)	32 (28–36)	33 (29–38)	28 (31–37)	0.403
Time spent in ED (minutes; 95% CI)	335 (316–354)	338 (317–360)	324 (285–367)	0.577
SRGS (median; IQR)	2 (1–3)	2 (1–3)	2 (2–3.5)	0.014
SRGS (*n*, %)				0.152
- Low risk (0–1)	50 (25.5)	43 (28.5)	7 (15.6)	
- Intermediate risk (2–4)	135 (68.9)	101 (66.9)	34 (75.6)	
- High risk (≥ 5)	11 (5.6)	7 (4.6)	4 (8.9)	
NEWS2 (*n*, %)				0.255
- Low risk (0–4)	142 (72.4)	111 (73.5)	31 (68.9)	
- Medium risk (5–6)	22 (11.2)	16 (10.6)	6 (13.3)	
- High risk (≥ 7)	32 (16.3)	24 (15.9)	8 (17.8)	
D-dimer (mcg/dL)	1972 (1679–2315)	1572 (1349–1832)	4252 (2840–6366)	< 0.001

Abbreviations: CI, confidence interval; CTPA, computed tomography pulmonary angiography; ED, emergency department; IQR, interquartile range; NEWS2, National Early Warning Score 2; PE, pulmonary embolism; SRGS, simplified revised Geneva score

^∗^ *p* value by ANOVA; *χ*^2^ test or *χ*^2^ for linear trends when indicated.

## Data Availability

The data that support the findings of this study are available from the corresponding author upon reasonable request.
